# Antibacterial and Immunostimulatory Effects of Raziz Date Palm Pits in *Streptococcus agalactiae*-Infected Red Hybrid Tilapia

**DOI:** 10.3390/biology14101356

**Published:** 2025-10-03

**Authors:** Disha Varijakzhan, Chou-Min Chong, Annie Christianus, Aisha Abushelaibi, Swee-Hua Erin Lim, Wan-Hee Cheng, Eakapol Wangkahart, Kok-Song Lai

**Affiliations:** 1 Institute of Bioscience, Universiti Putra Malaysia, Serdang 43400, Selangor, Malaysia; dishavarijakzhan@gmail.com; 2 Laboratory of Sustainable Aquaculture (Aqualab), International Institute of Aquaculture and Aquatic Sciences (I-AQUAS), Universiti Putra Malaysia, Port Dickson 71050, Negeri Sembilan, Malaysia; annie@upm.edu.my; 3 Department of Aquaculture, Faculty of Agriculture, University Putra Malaysia, Serdang 43400, Selangor, Malaysia; 4 Al Dhaid University, Al Sidra, Al Dhaid P.O. Box 14130, Sharjah, United Arab Emirates; AAbuShlaibi@sharjah.ac.ae; 5 Health Sciences Division, Abu Dhabi Women’s College, Higher Colleges of Technology, Abu Dhabi P.O. Box 41012, United Arab Emirates; lerin@hct.ac.ae (S.-H.E.L.); lkoksong@hct.ac.ae (K.-S.L.); 6 Faculty of Health and Life Sciences, INTI International University, Persiaran Perdana BBN, Putra Nilai, Nilai 71800, Negeri Sembilan, Malaysia; wanhee.cheng@newinti.edu.my; 7 Laboratory of Fish Immunology and Nutrigenomics, Applied Animal and Aquatic Sciences Research Unit, Division of Fisheries, Faculty of Technology, Mahasarakham University, Khamriang Sub-District, Kantarawichai 44150, Maha Sarakham, Thailand; eakapol.w@msu.ac.th

**Keywords:** antibacterial activity, date palm pits, methanol extract, red hybrid tilapia, *Streptococcus agalactiae*

## Abstract

Aquaculture is an important food sector, which involves the commercial production of fish for consumption. Tilapias (*Oreochromis* sp.) are hardy and are one of the most commonly produced fishes in the aquaculture industry. Disease outbreaks caused by *Streptococcus agalactiae*, however, widely affect tilapia farms, resulting in high mortality. Consequently, this may lead to the misuse of antibiotics for the prevention of disease or overuse of antibiotics when used for the treatment of fishes, contributing to antibiotic resistance. In this study, date palm pits, a waste product from the date palm industry, were tested for potential antibacterial activity against *S. agalactiae* and for their ability to act as an immune enhancer in vitro through the use of the head kidney and serum from healthy adult tilapias. An in vivo study was performed by dividing tilapias into two groups, consisting of infected *S. agalactiae* and uninfected *S. agalactiae*. Each group consisted of extract-fed and distilled-water-fed tilapia. Then, the serum, spleen and head kidney were isolated from both groups and tested for their respiratory burst, lysozyme and myeloperoxidase activities. The results from this study indicate that the Raziz methanol extract at a concentration of 1 g/mL inhibited the growth of *S. agalactiae*, and concentrations of 10 mg/mL, 2 mg/mL and 0.016 mg/mL displayed the highest respiratory burst, lysozyme and myeloperoxidase activities, respectively, in vitro. In the infected group, extract-fed tilapias showed a significant effect on respiratory burst activity and lysozyme activity compared to the distilled-water-fed tilapias, while no significant activity was observed in the uninfected group. In conclusion, the Raziz methanol extract has promising potential to act as an antibacterial agent, and it enhanced the innate immune function during active infection of *S. agalactiae*.

## Simple Summary

Aquaculture is an important food sector that produces fishes commercially for consumption. A major constraint faced by farmers in aquaculture is disease outbreak, which impacts them financially. Tilapia is a species that is highly farmed, and disease outbreak by *Streptococcus agalactiae* results in major economic losses for farmers. Hence, to reduce mortality and improve the immune system of tilapia, date palm pits, a waste product from the date palm industry, were screened for antibacterial properties against *S. agalactiae*, as well as for their ability to enhance the immune system of red hybrid tilapia (*Oreochromis* sp.). The methanol extract of Raziz date palm pits indicated potential antibacterial activity, with the ability to act as an immune enhancer in both in vitro and in vivo immune assays.

## 1. Introduction

Aquaculture is an important sector in the food industry. It is a common source for the commercial production of fishes while simultaneously aiding in the replenishment of wild stock in natural habitats. According to the Food and Agriculture Organization of the United Nations, there was a surge in overall global fish production in 2022, 223.2 million tons, while the aquaculture industry also experienced an increase in production, of up to 130.9 million tons (59%) [1].

One of the major issues faced by farmers in the aquaculture industry is disease outbreak. For instance, Streptococcosis is a disease that widely affects tilapia (*Oreochromis* sp.) farming, with a mortality rate of 60–70% [2,3]. Globally, disease causes annual losses of USD 1–9.6 billion in finfish aquaculture, while Streptococcosis in tilapia alone accounts for an estimated USD 150 million in economic losses [4]. The causative agent of Streptococcosis is a Gram-positive bacterium, *Streptococcus agalactiae*, which can be further categorized into ten different serotypes based on serotype-specific antigens and categorized as zoonotic in nature [5,6,7]. The serotypes are Ia, Ib, II, III, IV, V, VI, VII, VIII, and IX [8]. Serotypes Ia, Ib, II, and III are the major cause of infection in fishes, whereas serotypes Ia, II, III and V are common causative agents in humans [6,7,9].

The common treatment for *S. agalactiae* infection in humans and fishes is antibiotic therapy, with vaccination as a preventive measure [2,6]. Ampicillin and penicillin G are the first line of therapy for infections caused by *S. agalactiae* [6]. Antibiotics, such as erythromycin, clindamycin, vancomycin, ciprofloxacin, chloramphenicol and tetracycline, are used as alternative antibiotics for those allergic to penicillin and are also commonly used in the aquaculture industry [10,11,12,13]. As the treatment for *S. agalactiae* infection is highly dependent on these antibiotics, a significant number of cases reporting increased antibiotic-resistant and residue-related issues has been reported as a consequence [13,14,15,16,17].

In the past, plants and their products have been widely explored for various bioactivities such as antimicrobial activities against various pathogenic bacteria and enhancing immune systems [18,19]. For instance, the date palm (*Phoenix dactylifera*) and its products such as its pits, a waste product, were reported to exhibit significant antimicrobial activity, while the fruit was shown to stimulate the cellular immune system [18,20]. Date pits, widely produced by the date palm processing and packaging industries, are widely utilized as a feed material for poultry and animals because they are rich in natural antioxidants and bioactive polyphenols [18,21]. Further, they are also known to improve white blood cell counts and act as an anti-inflammatory agent by down-regulating the expression of pro-inflammatory mediators [22,23,24]. Date palm pits in powder form have been extensively explored as a fish feed and were found to improve growth and enhance the fish immune system in general, whilst reducing the cost of making fish feed, sustaining the aquaculture industry [25,26]. However, the utilization of a particular extract of date pits as a part of fish feed as an antibacterial and immune enhancer has not been explored widely, along with its effects at the cellular level during active infection. In addition to benefits such as a reduction in cost in the development of fish meal and a reduction in antibiotic residue production along with minimizing antibiotic misuse, the outcome from this study can aid in further explaining the effect date palm pits have on tilapia at the cellular level during an active infection phase.

In this study, we determined the antibacterial activity of various date pit cultivars from the United Arab Emirates against *S. agalactiae*. In addition, a date pit extract with better antibacterial ability was tested for the ability to act as an immune enhancer in infected adult red hybrid tilapias.

## 2. Materials and Methods

### 2.1. Collection of Date Palm Pits

Date palm pits of varieties Bouman, Fardh, Khalas, Lulu, and Raziz that are commercially produced and available in United Arab Emirates were kindly provided by Mr. Altayeb Abd Alhay Mohamed, from Flag Holdings based at Al-Ain, Abu Dhabi, UAE. The pits were washed and sun-dried. Then, the dried pits were ground using a grinder (Preethi Blue Leaf Gold MG150, 750 Watts). The fine powder was filtered using a strainer and stored in a container.

### 2.2. Preparation of Date Pit Extracts

Methanol extraction was performed by dissolving the fine Raziz date pit powder in methanol solvent following the maceration technique, as described by Bitwell et al. [27]. About 20 g of powdered date pits was soaked in 120 mL methanol and kept in a shaker incubator for 4 days at 30 °C, 200 rpm. Then, the mixture was filtered using Whatman No.1 Filter paper with an interval of 2 days. The collected extract was concentrated using a rotary evaporator at 40 °C with a vacuum pump. Then, the extract was left at 40 °C in an incubator to dry. The dried extract was stored at −20 °C for further analysis. The process was repeated using chloroform and water to obtain their respective extracts. The procedure was repeated for Bouman, Fardh, Khalas and Lulu date pit powder.

### 2.3. GCMS Analysis

Compounds present in the Raziz methanol crude extract (RDPME) were analyzed using the Agilent 7890 gas chromatograph (GC), coupled to Agilent 5975 quadruple mass detector (MS), and HP-5MS capillary column (30 m × 250 µm × 0.25 µm) was used (Agilent Technologies, Santa Clara, CA, USA). The carrier gas in the MS was helium, with a flow rate of 1 mL/min. The methanol crude extract was injected into the GC injection port with a 1:20 split mode. The temperature of the oven was set at 50 °C, followed by a gradual increase in the temperature at a rate of 10 °C per minute for 3 min until it reached 280 °C. The ion source and transfer line temperatures were set at 220 °C and 280 °C, respectively. The electron impact mass spectra were recorded at 70 eV, and the compounds were identified using MSD Chemstation Enhanced Data Analysis Software (E.02.02.1431 version, Agilent Technologies) and the National Institute of Standards and Technology (NIST 20) database. The analysis was performed in triplicate.

The compounds which are commonly found in the triplicates are presented by calculating the relative percentage from their GC peak area.

### 2.4. Bacterial Strain and Growth Condition

The bacterial strain *S. agalactiae* was isolated from an organ of infected tilapia (weight: 0.07–0.09 kg, length: 12–15 cm), from a fish farm located at Semenyih, Malaysia. The isolated bacteria were confirmed via polymerase chain reaction (PCR). Primers used were based on 16S ribosomal RNA, and the sequence obtained was confirmed as *S. agalactiae* via BLAST (NCBI, BLAST + 2.10.0 version) (Supplementary Figure S1). Further confirmation of serotype was performed by targeting *cps* genes. The list of primers and condition of PCR are listed in Supplementary Table S1. The strain was cultured and maintained on Brain Heart Infusion Agar (BHI; Sigma Aldrich, St. Louis, MO, USA). For subsequent tests, a single colony was inoculated into Mueller–Hinton (MH; Sigma-Aldrich, St. Louis, MO, USA) broth for disc diffusion assay at 37 °C, shaking at 200 rpm for 18 h.

### 2.5. Antibiotics’ Efficacy Determination

As the *S. agalactiae* bacterial strain was isolated from an infected tilapia, the strain was tested for sensitivity towards commonly used antibiotics, such as ampicillin, tetracycline and chloramphenicol, as detailed in the CLSI M100 [28]. The test was performed in triplicate (Supplementary Table S2: Concentration of antibiotics tested and analyzed for the expected zone of inhibition (ZI); Table S3: Result of ZI of commercial antibiotics tested against *S. agalactiae*).

### 2.6. Antibacterial Activity

Antibacterial activity of *S. agalactiae* against respective extracts of Raziz date pits was determined by using the agar disc diffusion method, as detailed in CLSI M2-A9 [29]. Methanol and chloroform crude extracts of 2 g/mL, 1 g/mL, and 0.5 g/mL were prepared, respectively, whereas 8.33%, 16.65% and 33.3% were prepared for water extract. About 10 µL of the extract was impregnated into sterile 6 mm (diameter) blank discs. The positive control used was ampicillin antibiotics at 20 µg/mL concentration. The assay was conducted in triplicates, and the plates were incubated at 37 °C for 20 h. The antibacterial activity was determined by measuring the diameter of the ZI around the disc. The results were the average of the triplicates and indicated as ZI (mm) ± standard error of mean (mm). The experiment was repeated for methanol, chloroform and water extracts of Bouman, Fardh, Khalas and Lulu.

### 2.7. In Vitro Immune Assays 

#### 2.7.1. Leucocyte Suspension Collection

Three healthy adult red tilapias from a fish farm at Agriculture Faculty, Universiti Putra Malaysia (average weight: 0.262 ± 0.097 kg; size: 23.62 ± 1.94 cm), were collected and quarantined for 7 days to ensure they were in healthy condition. Then, these healthy tilapias were anaesthetized using MS-222 (Sigma-Aldrich, St. Louis, MO, USA) at a concentration of 100 mg/L. Blood was drawn from the caudal area and allowed to form clots. The serum was collected via centrifugation at 2000 rpm for 10 min at 4 °C. The serum was transferred into sterile 1.5 mL Eppendorf tubes and stored at −20 °C for further analysis.

The head kidney was dissected from the tilapia. The organ was meshed and filtered through a 40 µm nylon mesh with the addition of phosphate buffer saline (PBS). The resultant pellet was transferred into a sterile Eppendorf tube and centrifuged at 2000 rpm, for 5 min. Pellets obtained were mixed with 1 mL freezing media (Gibco Dulbecco’s Modified Eagle Medium (Thermo-Fisher Scientific, New York, NY, USA) + 10% fetal bovine serum (Capricorn Scientific, Ebsdorfergrund, Germany) + 10% DMSO). Cell counts were performed using a hemocytometer via trypan blue as follows:

$$\mathrm{Cell}\;\mathrm{viability}\;(\%)\;=\;\frac{\mathrm{Number}\;\mathrm{of}\;\mathrm{viable}\;\mathrm{cells}}{\mathrm{Total}\;\mathrm{cells}\;\mathrm{counted}}\times 100\%$$

$$\mathrm{Concentration}\;(\mathrm{cells}/\mathrm{mL})=\frac{\mathrm{Total}\;\mathrm{cells}\;\mathrm{counted}\;\mathrm{in}\;4\;\mathrm{squares}}{4}\times \mathrm{Dilution}\;\mathrm{factor}\times 10^{4}$$

where

$$\mathrm{dilution}\;\mathrm{factor}=\frac{\mathrm{Final}\;\mathrm{volume}\;\mathrm{of}\;\mathrm{solution}}{\mathrm{Volume}\;\mathrm{of}\;\mathrm{cell}\;\mathrm{suspension}}$$

The single-cell suspension consisted of about 98% viable cells and contained 5 × 10^6^ cells/mL. The suspension was stored at −80 °C for further analysis.

The concentrations of the extracts tested were 50 mg/mL, 10 mg/mL, 2 mg/mL, 0.4 mg/mL, 0.08 mg/mL and 0.016 mg/mL. The concentration of the cell suspension tested was 1 × 10^6^ cells/mL, for all the assays.

#### 2.7.2. Lysozyme Assay

Lysozyme activity (LA) of the serum was determined via lysis of Gram-positive bacteria *Micrococcus lysodeikticus* (Sigma-Aldrich, St. Louis, MO, USA), as detailed in [30]. A volume of 150 µL of 0.02% *M. lysodeikticus* suspension (0.20 mg/mL in 0.1 M sodium phosphate buffer, pH = 6.2) was mixed with 50 µL serum leucocyte suspension and 50 µL of extract in a microtiter plate. The plate was immediately placed in the ELISA reader at an optical density of 570 nm and recorded as A1 (0 min). The plate was then incubated at 37 °C for 30 min. Then, it was transferred to an ice bath to stop the reaction and was read again at 570 nm (A2). The assay was performed in triplicate. The control for this assay was without the addition of *M. lysodeikticus* suspension.

Lysozyme activity (U) was calculated using the formula:

Lysozyme activity (U) = (A1 − A2)/A1

#### 2.7.3. Myeloperoxidase Assay

Myeloperoxidase is an enzyme that can be found in azurophilic granules of neutrophils. The activity of myeloperoxidase enzyme (MPO) was determined as per the procedure in [31] with slight modification. The assay was performed by adding 15 µL of head kidney leucocyte suspension and 15 µL of extract to the wells of a microtiter plate. Then, 135 µL Hank’s Balanced Salts (HBSS; Capricorn Scientific, Ebsdorfergrund, Germany) (background) was added, followed by 45 µL of 20 mM 3,3′,5,5′-tetramethylbenzidine hydrochloride (TMB; Sigma-Aldrich, St. Louis, MO, USA) and 45 µL of 5 mM hydrogen peroxide. The plate was incubated at 37 °C for 2 min, and the reaction was stopped by adding 53 µL of 4 M sulphuric acid. The above steps were repeated with 0.02% cetyltrimethylammonium (CTAB) (lysed) and 50 nM phorbol myristate (PMA in DMSO; Sigma-Aldrich, St. Louis, MO, USA) (stimulated), replacing HBSS. The plate was read at 450 nm using a microplate reader. The assay was performed in triplicate. Control of the assay consists of a leucocyte suspension and crude extract.

The percentage of MPO (MPO%) released was calculated using the formula:

$$\mathrm{MPO}\%\;=\;\frac{\mathrm{OD}\;\mathrm{stimulated}\;-\;\mathrm{OD}\;\mathrm{background}}{\mathrm{OD}\;\mathrm{lysed}\;-\;\mathrm{OD}\;\mathrm{background}}\times 100\%$$

#### 2.7.4. Respiratory Burst Assay

The assay was performed as detailed in [32], where zymosan (Sigma-Aldrich, St. Louis, MO, USA) was used as a stimulant. A volume of 100 µL head kidney leucocyte suspensions, 100 µL zymosan (5 mg/mL) and 100 µL extract was deposited in microtiter plate wells and allowed to react at room temperature for 30 min. Then, the zymosan was discarded via centrifugation at 2000 rpm for 5 min. The leucocytes were washed three times with PBS, followed by the addition of 100 µL of nitroblue tetrazolium (NBT; 2 mg/mL; Sigma-Aldrich, St. Louis, MO, USA) into each well and allowed to react for 30 min at room temperature. The reaction of NBT was stopped by adding 100 µL of 100% methanol, followed by 120 µL of 2M potassium hydroxide and 140 µL DMSO. The respiratory burst activity (RBA) was measured in triplicate at an optical density of 540 nm using an ELISA reader. The assay was repeated without the addition of zymosan for basal activity. The assay was performed in triplicate. RBA was calculated using the formula:

RBA = Stimulated Activity (with zymosan stimulation) − Basal Activity (without zymosan stimulation)

### 2.8. S. agalactiae Infection in Adult Red Hybrid Tilapia

#### 2.8.1. Experimental Set-Up

The experiment was conducted in two groups, where a total of 12 glass aquarium tanks (90 cm × 30 cm × 45 cm) were used. All tilapias were purchased from Ladang Taman Pertanian, Universiti Putra Malaysia, Puchong, and consist of combination of adult males and females. A total of 200 red hybrid adult tilapias were purchased, and each tank was randomly allocated 16–17 tilapias. Each aquarium tank was filled with 70 L of water, where the water was replaced every two days. Water quality of each aquarium was monitored daily using a Multiprobe Water Quality Meter (YSI 556 Multiprobe, Yellow Springs, Ohio, USA). The parameters were maintained as followed: (i) temperature: 27–28 °C; (ii) dissolved oxygen: 4.5–4.8 mg/L; (iii) pH: 7.5–7.7; and (iv) ammonia–nitrogen: ≤0.01 mg/L. All the tilapias were fed using commercial pellet twice daily and were acclimatized for a duration of 14 days, prior to feeding trial.

##### *S. agalactiae* Culture

*S. agalactiae* was prepared for bacterial challenge study, as described in [33,34], with slight modifications. An overnight culture of *S. agalactiae* was utilized and grown in Mueller–Hinton broth at 37 °C in an incubator shaker at 200 rpm for 18 h. A McFarland Standard 10.0 was prepared, as detailed in [35], as a reference for a concentration of 3.0 × 10^9^ cells/mL. The fresh culture was centrifuged at 10,000 rpm for 5 min, and supernatant was discarded. The pellet was resuspended in 0.85% saline to a concentration of 3.0 × 10^9^ cfu/mL by comparing with the McFarland Standard. Freshly prepared culture was also plated on Mueller–Hinton Agar to determine the concentration via plate count method.

##### Infection of *S. agalactiae*

The first group consisted of 100 adult red tilapias (0.1263 ± 0.0053 kg) assigned to 6 glass aquarium tanks, which are designated as the “Uninfected group”. Randomly, 3 aquarium tanks were selected to be fed with 0.1 mL of 1 g/mL RDPME (Con-RD), and another 3 tanks were fed with 0.1 mL distilled water (Con-DW) via oral gavage for 14 days, without *S. agalactiae* infection.

The second group consisted of 100 adult tilapias (0.09442 ± 0.0049 kg ) assigned to 6 glass aquarium tanks and are designated as “Infected group”. Randomly, 3 aquarium tanks were selected to be fed with 0.1 mL of 1 g/mL RDPME (Inf-RD), and another 3 tanks were fed 0.1 mL distilled water (Inf-DW) via oral gavage after completion of acclimatization period. Firstly, for 5 days, the tilapias were fed with RDPME and distilled water prior to infection, followed by 10 days post-infection with *S. agalactiae*. The tilapias were infected with *S. agalactiae* via submersion method, as detailed in [36]. Prior to infection, the water in each aquarium tank was half emptied. Then, *S. agalactiae* was prepared from an overnight culture; it was adjusted to achieve a final concentration of 5.9 × 10^8^ cfu/mL. The tilapias were left in this condition for 30 min, and then the aquarium tank was topped up with water to 70 L.

Post-infection, the tilapias from both groups were observed for symptoms and or mortality. All treatments were performed in triplicate. Subsequently, on Day 11 post-infection, two fishes from each tank of the “Infected group” and “Uninfected group” were randomly selected and euthanized using Transmore (Nika, Ipoh, Perak, Malaysia). Samples of blood, spleen, head kidney, and intestine were collected for further analyses.

#### 2.8.2. In Vivo Immune Assays

The collection of serum from blood, leucocyte suspension from head kidney and spleen of each tilapia was performed, as detailed in Section 2.7.1. Leucocytes collected were further analyzed for lysozyme assay, myeloperoxidase assay and respiratory burst assay, as detailed in Section 2.7.2, Section 2.7.3 and Section 2.7.4, respectively.

#### 2.8.3. Bacterial Load Assay

A bacterial load assay was performed on the infected group, as detailed in [33], with slight modifications. Spleen and intestine were harvested from both Inf-RD and Inf-DW, where small sections of spleen and intestine were isolated and transferred into a sterile phosphate buffer solution (PBS). The sample was vortexed, and a volume of 100 µL of the solution was plated onto a BHI agar. The plates were then incubated at 37 °C for 20 h, to observe bacterial growth.

### 2.9. Statistical Analysis

All the data generated were analyzed using Minitab version 19.0 (Minitab, LLC, State College, PA, USA). The normality of the data was determined using Shapiro–Wilk test. The equality of variance was determined using Levene’s Test. One-way ANOVA was used to analyze the significance of the groups, when the data are normally distributed. For the post hoc analysis, Tukey test was performed for parametric data of equal variance samples, whereas for non-parametric data, post hoc analysis with Mann–Whitney was carried out.

## 3. Results

### 3.1. Antibacterial Activity of Date Palm Pit Extracts

All five cultivars’ (Bouman, Fardh, Khalas, Lulu and Raziz) methanol, chloroform and water extracts were tested for antibacterial activity against *S. agalactiae*. Table 1 below summarizes the antibacterial activity of Raziz methanol extract (RDPME), whereas Supplementary Table S4 showcases the antibacterial activity of all five cultivars and their respective extracts. RDPME exhibited a strong antibacterial activity against *S. agalactiae* at a lower concentration of 1 g/mL compared to other extracts and was found to be significant compared to the control (Ampicillin, 20 µg/mL).

### 3.2. GC-MS Analysis

As the RDPME was discovered to have potential antibacterial activity against *S. agalactiae*, the extract was analyzed for the compounds responsible for the activity. Table 2 below summarizes the compounds majorly identified in the extract. Based on the analysis, the compounds are majorly made up of fatty acids, such as oleic acid (23.96%), dodecanoic acid (14.73%), n-hexadecanoic acid (7.91%) and tetradecanoic acid (7.78%).

### 3.3. In Vitro Immune Assays

#### 3.3.1. Lysozyme Assay

**Figure 1:**
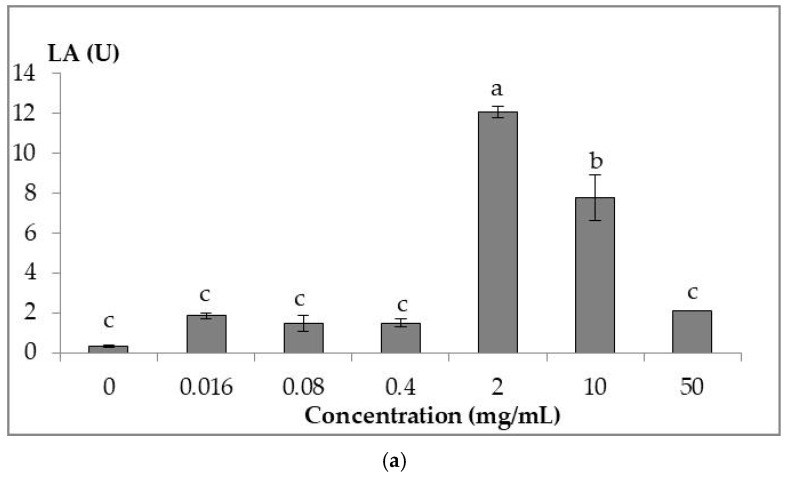

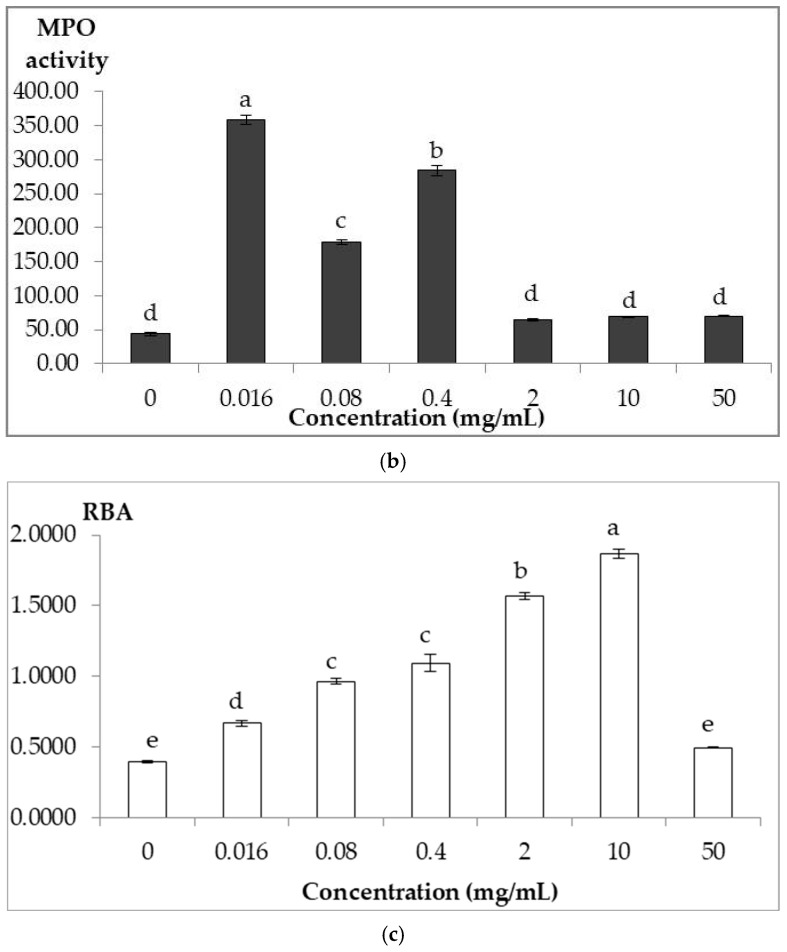
In vitro immune assays: lysozyme assay (**a**), myeloperoxidase assay (**b**), and respiratory burst assay (**c**) against RDPME across 5-fold concentrations. (**a**) LA against RDPME, where different letters indicate significance difference via Tukey’s test compared to control (*p* < 0.05); (**b**) MPO activity against RDPME, where different letters indicate significance difference via Tukey’s test compared to control (*p* < 0.05); (**c**) RBA against RDPME, where different letters indicate significance difference via Tukey’s test compared to control (*p* < 0.05).

Figure 1a shows the lysozyme activity of serum collected from healthy adult tilapias. From the Figure, RDPME at a concentration of 2 g/mL showed the highest activity, followed by 10 mg/mL, and it is found to be significant compared to the control (*p* < 0.05) (absence of RDPME).

#### 3.3.2. Myeloperoxidase Assay

Figure 1b showcases the activity of myeloperoxidase enzyme. From the graph, the highest MPO% was found to be at 0.016 mg/mL RDPME, followed by 0.4 and 0.08 mg/mL, respectively. As the concentration of the extract increased further, the activity was observed to be higher compared to the control, but not statistically significant, where concentrations of 2, 10 and 50 mg/mL remained constant.

#### 3.3.3. Respiratory Burst Assay

Figure 1c shows RBA in the presence of RDPME at different concentrations. From the graph, RDPME at a concentration as low as 0.016 mg/mL exhibited significant activity compared to the control (*p* < 0.05). The activity exhibited an increasing trend, where the highest activity was recorded at a concentration of 10 mg/mL of RDPME.

### 3.4. In Vivo Study of RDPME Effect on Tilapia

#### 3.4.1. In Vivo Immune Assay

##### Lysozyme Assay

**Figure 2:**
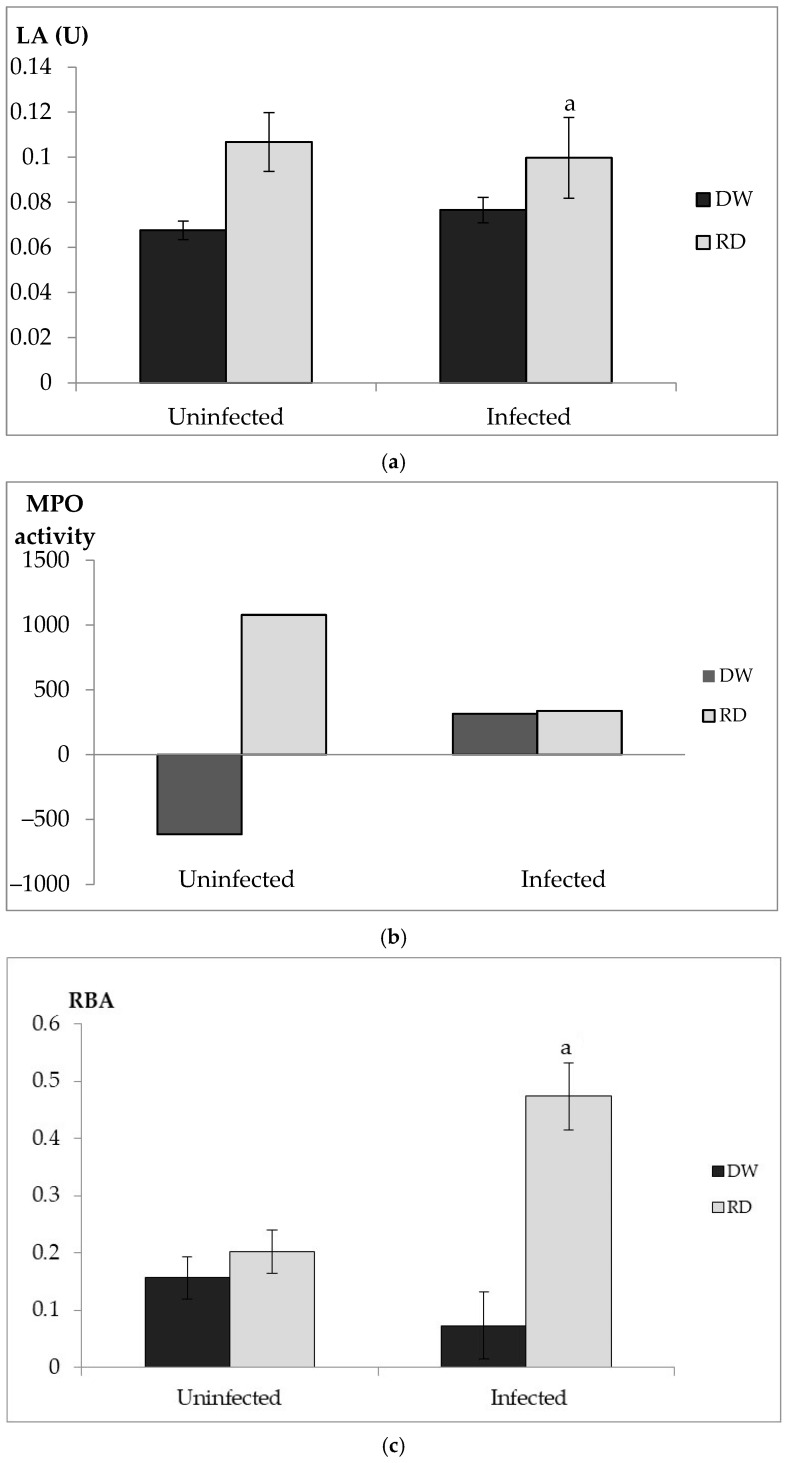
In vivo immune assays: lysozyme assay (**a**), myeloperoxidase assay (**b**), and respiratory burst assay (**c**) of infected and uninfected group in both DW/RD. (**a**) LA with letters indicates significant differences via Tukey’s test (*p* < 0.05). LA in RD fed is not significant compared to DW fed in the absence of infection; (**b**) MPO activity is not significant (*p* ≥ 0.05) via Tukey’s test for samples obtained from head kidney during and in absence of infection; (**c**) RBA of tilapia fed with RDPME during infection and in absence of infection from head kidney. RBA with letters indicates significant differences via Tukey’s test (*p* < 0.05). RD: RDRME fed; DW: distilled water fed.

Figure 2a shows the LA of serum isolated from *S. agalactiae*-infected tilapia and in the absence of active infection. From the data, the extract has been shown to be able to enhance the LA compared to tilapias not treated with the extract in both groups. Moreover, a significant effect by the extract (*p* < 0.05) was observed during an infection.

##### Myeloperoxidase Assay

Figure 2b shows the myeloperoxidase activity isolated from the head kidney of *S. agalactiae*-infected tilapias and tilapias in the absence of active infection. The data show that during the infection phase, there is no significant MPO activity in both DW- and RD-treated fish. However, the RD-treated fish in the uninfected group showcases an increase in MPO.

##### Respiratory Burst Assay

Figure 2c shows the RBA of leucocytes isolated from the head kidney from both infected and uninfected groups. RBA was significantly higher in RD-treated fish compared to the DW-treated fish (*p* < 0.05) during active infection of *S. agalactiae*. However, there was no significant RBA in the uninfected group in both DW/RD-treated fish.

#### 3.4.2. Bacterial Load Assay

**Figure 3:**
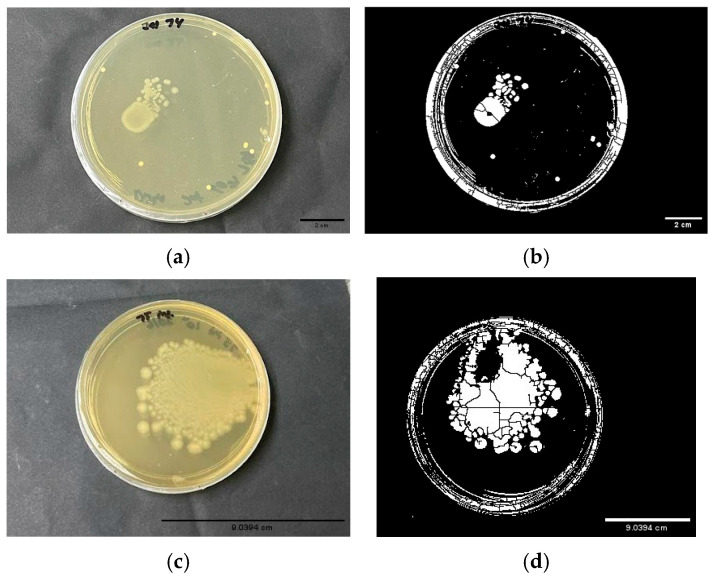
The bacterial load assay from intestine of *S. agalactiae*-infected tilapia. (**a**) Bacterial growth observed in BHI agar from intestine sample isolated from Inf-RD; (**b**) J-image result calculating the percentage area covered on the plate by the bacterial growth. From the J-image, the bacterial growth only covered 5.101% of the plate. (**c**) Bacterial growth observed on BHI agar from intestine sample isolated from Inf-DW; (**d**) J-image result calculating the percentage area covered on the plate by bacterial growth. Based on the image, 30.847% of the area was covered with bacterial growth.

Bacterial load assay was performed in the infected tilapia group. Intestine and spleen samples were tested for bacterial growth. There was no bacterial growth observed in spleen samples from both Inf-RD and Inf-DW. However, bacterial growth was observed in intestine samples from both Inf-RD and Inf-DW. Figure 3 below depicts the growth of the bacteria, where 3(a,b) belong to the intestine from Inf-RD, with the bacterial growth covering 5.101% of the area of the plate, whereas there is more prominent bacterial growth in the intestine sample from Inf-DW 3(c,d) where bacterial growth covered 30.847% of the area. The average of bacterial growth was tested for significance via a *t*-test. The outcome of the test showed a *p*-value of 0.004 (*p* < 0.05), which indicates feeding the extract to tilapia during active infection of *S. agalactiae* had an association in reducing the bacterial load, compared to DW-treated fish.

## 4. Discussion

*S. agalactiae* isolated from infected tilapias was tested for its antibiotic sensitivity. The bacterial strain was found to be resistant to ampicillin at a dosage of 0.25 µg/mL, which is categorized as sensitive in the CLSI M100 guideline [28]. The strain was also observed to be resistant to chloramphenicol and tetracycline (Supplementary Table S3). The finding of resistance towards ampicillin may suggest the rise of antibiotic resistance, as ampicillin is the first line of antibiotics. Hence, there is great urgency to identify alternative solutions for the treatment of *S. agalactiae* infection. Our results for the antibiotic susceptibility test of *S. agalactiae* are in accordance with studies conducted in Hong Kong and South Africa for the bacterial strain isolated from fish and pregnant women, respectively [12,37]. In both cases, the strain was non-susceptible towards penicillin and gentamicin but resistant towards erythromycin, tetracycline, clindamycin, chloramphenicol and ciprofloxacin [12,37].

Of all the extraction solvents (chloroform, methanol and water) tested, the methanol extract of Raziz was chosen in this study because it displayed the highest antibacterial activity compared to the other solvent extracts (Supplementary Table S4). The methanol extract exhibited weak antibacterial activity at a concentration as low as 0.5 g/mL, whereas the chloroform extract exhibited weak antibacterial activity at the highest concentration of 2.0 g/mL. Similarly, the water extract exhibited weak antibacterial activity at the highest concentration of 33.3%. The difference in the antibacterial activity observed among these extracts is due to the type and polarity of solvents used [38]. Generally, a polar solvent such as methanol has the ability to extract various compounds with phytochemical activities [18,38]. It has been shown that the extraction using polar solvents contains compounds with higher antioxidant activity, reducing properties and free radical scavenging activity compared to the non-polar solvents [39,40]. For instance, gallic acid, a phenolic acid compound, was found to be in abundance in the methanol extract of Ajwa date pits [41]. Gallic acid was found to be more effective against Gram-positive bacteria, i.e., *Staphylococcus aureus* and *Streptococcus* sp., compared to Gram-negative bacteria at a concentration of 10 µg.

In this study, the highest concentration tested was 2.0 g/mL because the crude extract was not able to dissolve in water as the concentration increased. As the concentration increased beyond 2.0 g/mL, an extract paste was formed, hindering its impregnation on the disc for the antibacterial assay. Nonetheless, a 2.0 g/mL concentration is adequate to show great antibacterial activity against *S. agalactiae*.

In a study conducted on various date pits, Khalas extracts (ethyl acetate and acetone/water) and Khodari extracts (ethyl acetate and hexane) were able to inhibit *S. aureus* and *E. coli* [42]. In addition, Abu Mann date pits, acetone/water, ethyl acetate and methanol/chloroform extracts were also reported to significantly inhibit the activity of *S. aerues* and *E. coli*; the methanol/chloroform extract was found to be more potent against *E. coli* compared to the other extracts. Similar results were also reported for Ajwa date pits acetone/water, methanol/chloroform and ethyl acetate extracts [42].

The antibacterial activity of RDPME can be attributed to the presence of the different compounds in the extract. Based on our GCMS analysis, the extract was found to contain oleic acid, dodecanoic acid, n-hexadecanoic acid and tetradecanoic acid, which have been reported to have antibacterial activity against various Gram-positive and Gram-negative bacteria [43,44,45]. Oleic acid, an unsaturated fatty acid, was present in a high concentration in the methanol extract. The compound was found to be able to inhibit the FabI enzyme of *S. aureus* and *E. coli* by inhibiting the fatty acid synthesis of the bacteria [46]. Meanwhile, dodecanoic acid was reported to be more effective against Gram-positive bacteria such as *S. aurues* by disrupting the membrane [47].

The innate immune system in fishes is the first line of the defense mechanism to combat infections [48]. The innate immune system of tilapia can be evaluated via RBA, lysozyme activity and myeloperoxidase activity. Herbal extracts from various parts of plants, such as the methanol extract of *Terminalia catappa* leaves and aqueous extract of *Scutellaria baicalensis* roots, have been reported to effectively increase the innate immune system in tilapia [32,49]. Other than potential antibacterial activity, the RDPME exhibited the ability to enhance the immune system in vitro. From our study, we observed that the extract was able to improve the activities of lysozyme, myeloperoxidase and respiratory burst activity when compared to the control (without extract).

Respiratory burst is the release of reactive oxygen species (ROS) by neutrophils predominantly that is responsible for killing pathogens [50]. The assay measures the amount of superoxide produced via the reduction in nitroblue tetrazolium by the ROS. The RDPME at 50 mg/mL showed a sudden reduction in RBA, after recording the highest RBA at 10 mg/mL. The sudden decrease in the RBA at the highest RDPME concentration (50 mg/mL) may be attributed to feedback or regulatory suppression mechanisms, where excessive immune stimulation triggers negative feedback to prevent the over-activation of immune responses. Such immunological down-regulation has been reported in fish exposed to high levels of herbal bioactivities [51]. A study conducted on common carps fed with date palm seed for 60 days also reported higher RBA compared to the control with an increase in the RBA in a dose-dependent manner [52].

Similar to RBA, the lysozyme assay also showed an increase in LA when the serum leucocyte suspension was treated with the RDPME. The LA of the control (without date extract) was observed to be higher at 0 min, whereas the LA was recorded to increase after 30 min of incubation with the RDPME, thus exhibiting the ability of the extract to enhance the functionality of the lysozyme enzyme. When the activity (with stimulant *M. lysodeikticus*) is compared to the LA for 0.4 mg/mL without *M. lysodeikticus*, it was recorded that the extract was able to increase the LA in the absence of *M. lysodeikticus*.

In two different studies, *Saccharomyces cerevisiae* (yeast)-fermented date seed meal and *Aspergillus oryzae*-fermented date seed meal were fed to Nile tilapias. In both studies, the yeast-fermented date seed meal and *A. oryzae*-fermented date seed meal showed an increase in LA compared to the control with an increase in the white blood cell count, indicating improved immune function [53,54]. The increase in LA in the stimulated cells with the extract may indicate that there is an increase in the production of lysozyme from lysosomes [55].

The myeloperoxidase assay measures the myeloperoxidase enzyme, which is found abundantly in the azurophilic granules of neutrophils [56]. A similar result to our study was observed when the pits of the Deglet Nour variety were incorporated into the feed of gilthead sea bream fish and recorded higher peroxidase activity after 30 days of feeding compared to the control [57]. However, when a date palm fruit-incorporated feed was fed to European sea bass, there was no significant peroxidase content observed [58]. This indicates that the source of the feed, the fish species and other factors such as the type of solvents used, condition during harvest and geographic location of a plant can affect the biological activities [19,59].

The ability of RDPME to improve the activities of respiratory burst, lysozyme and increasing myeloperoxidase content could be due to the presence of a mixture of fatty acids. Fatty acids have been reported to have various roles in the innate immune system of humans. For example, unsaturated fatty acids have been reported to increase the phagocytic activity of macrophages and increase ROS production and phagocytosis in neutrophils [60]. The ability of the fatty acids to induce an immune response depends on the number and position of double bonds as well as the carbon chain length [61,62]. As RDPME was found to consist of a mixture of fatty acids, it might have affected the RBA, lysozyme activity and MPO%.

As the in vitro study showcased the ability of RDPME to improve the activities of the innate immune system, it was necessary to examine the capability of the extract in a living organism, adult red hybrid tilapia, to assess the safety and toxicity of the extract in a tilapia and also to study the effect of the extract on an infected tilapia, to resemble an aquaculture farm environment. Following that, the RDPME was tested for in vivo effectiveness and toxicity. Healthy adult tilapias were fed with the extract for a duration of 14 days. During the time period, there was no mortality or physical sign of toxicity observed in tilapias.

Following the in vivo test, the tilapias exhibited symptoms of infection, such as cloudy eyes and yellowish color on the skin; however, no mortality was recorded in both the Inf-RD and Inf-DW groups. This may be because the tilapias were infected in a controlled laboratory setting, as various studies have showed that the virulence factors of *S. agalactiae* are influenced by stress factors such as over-crowding and high temperature [36,63,64,65]. LA and RBA were significant in their respective activities with the RD-treated fish. However, the uninfected group showed that there is no significant effect when the extract is fed in the absence of infection, having similar activity with DW-treated fish. During active *S. agalactiae* infection in tilapia, RD-treated tilapias showcased improved immune activities, compared to the control, indicating the ability of the extract to act as an immune enhancer. The uninfected group of MPO in Figure 2(b) of the head kidney exhibited a negative reaction value, which might be due to the lower response of phorbol myristate (PMA) in the absence of infection. PMA is a known artificial stimulus, which is capable of inducing neutrophil degranulation and ROS production [66]. However, in the absence of infection, the leucocytes are in a less responsive phase; thus, the oxidative burst capacity is low, resulting in a negative value compared to the MPO activity from infected samples.

Other than that, the intestine sample of RD-treated fish at a concentration of 1 g/mL showed a reduction in the bacterial load compared to the DW-treated fish. Similar to our date pit extract, there are few compounds from plant extracts such as carvacrol, cinnamaldehyde and capsicum in combination that were able to reduce the enteric pathogens in broiler chicken, simultaneous to developing innate immunity and reducing mortality [67]. The addition of the same mixture of plant extract also showed an improvement in intestinal health by improving microflora in the intestine, thus reducing the chances of diarrheal disease in pigs [67,68]. The ability of various extracts to improve the micro-flora in the gut is attributed to the various compounds present in the extracts such as tannins and saponins [69]. A similar result was also recorded among broiler chicken when dried dates and date pits were incorporated into respective diet meals, where pathogenic bacteria such as *Salmonella* sp., *Campylobacter* sp., *Shigella* sp., and *E. coli* were reduced [70,71].

One study conducted by incorporating marine algae (*Ulva fasciata*) extract on a Nile tilapia diet at a concentration of 100 mg/kg was able to stimulate lysozyme activity, phagocytic activity, and, in general, increase the count of white blood cells [72]. The authors attributed these activities to the presence of various bioactive compounds such as palmitic acid and oleic acid, which are also found in the RDPME. Other than that, date fruits, which are ¥-irradiated when incorporated into the diet of goldfish, showed improved ability in lysozyme activity, protease, alkaline phosphatase and Ig activities along with antibacterial activity on the skin of the goldfish against *Aeromonas hydrophilia* [73].

The ability of RDPME to present various bioactivities, such as antibacterial agent, immune enhancer and also the ability to reduce bacterial load during infection, is possibly due to the presence of various bioactive compounds. The presence of a mixture of saturated fatty acids comprising dodecanoic acid, tetradecanoic acid, and hexadecanoic acid has shown positive effects; oleic acid is the most abundant, so an unsaturated fatty acid will be able to enhance the immune system of tilapias. Oleic acid has been reported as an immune enhancer. It has shown the ability to induce an immune response in groupers exposed to *Vibrio vulnificus* by showing an increased response in lysozyme, respiratory burst and phagocytosis activities [74,75]. Similarly, using a rat as a model organism, oleic acid has been shown to increase the number of neutrophils found on the wounded region, where it was shown to be able to stimulate the production of interlukin-1 beta (IL-1β) [76]. Another study in rats treated with oleic acid-enriched olive oil showcased an increase in IL-8 production in the epithelial cells of the intestine, thus increasing cytokine secretion by monocytes and or macrophages, reducing the risk of infection [77]. Dodecanoic acid, the second-highest compound found in the extract, has also been shown to improve immune activity in swimming crab. The authors reported that feeding dodecanoic acid to the swimming crab enhanced the intestinal barrier and upregulated intestinal immune-related genes, such as *ppo*, *α2M*, *lys*, and *crustin*, and antioxidant-related genes, such as *Mnsod*, *cat*, *prx* and *trx* [78]. The authors also cited that the inclusion of dodecanoic acid in the diet showed a significant improvement in the microbiota composition of the swimming crab, thus reducing the bacterial load of *Vibrio*.

## 5. Conclusions

In conclusion, the methanol extract of Raziz date palm pits showed an ability to inhibit the growth of *S. agalactiae* and was able to improve the immune activities, such as lysozyme and respiratory burst activity, during active infection. These results indicate that the date palm pits can favorably enhance tilapia feed without any fear of antibiotic residuals in meat, reducing dependence on antibiotics to treat an infection.

## Figures and Tables

**Table 1:** Antibacterial activity of RDPME against *S. agalactiae*.

Concentration (g/mL)	Zone of Inhibition ^#^ (mm)
Control	20.222 ± 0.401
0.5	1.000 ± 1.00 ^b^
1	9.167 ± 0.441 ^a^
2	10.333 ± 0.333 ^a^

^#^ denotes the average zone of inhibition obtained from the triplicate, with standard error of mean. ^a,b^ *p*-value is significant compared to control (*p* < 0.05) via Tukey’s test.

**Table 2:** GCMS analysis of RDPME.

No.	ID	Compounds Classification	Average Composition (%)
1	Furfural	Aldehyde, Furan	0.54
2	4H-Pyran-4-one,2,3-dihydro-3,5-dihydroxy-6-methyl-	Saponin (Triterpene glycoside)	4.42
3	5-Hydroxymethylfurfural	Furan	6.07
4	Dodecanoic acid, methyl ester	Fatty acid	1.27
5	Dodecanoic acid	Saturated medium chain fatty acid	14.73
6	Tetradecanoic acid	Saturated long chain fatty acid	7.78
7	Hexadecanoic acid, methyl ester	Fatty acid	0.23
8	n-Hexadecanoic acid	Saturated long chain fatty acid	7.91
9	Oleic acid	Unsaturated fatty acid	23.96

## Data Availability

All the data will be available in the student’s thesis.

## Supplementary Materials

The following supporting information can be downloaded at: https://www.mdpi.com/article/10.3390/biology14101356/s1, Table S1: Primer sequences and conditions of PCR cycle; Figure S1: BLAST result of isolate indicating *S. agalactiae*; Table S2: Concentration of commercial antibiotics tested and expected zone of inhibition; Table S3: Zones of inhibition for commercial antibiotics tested; Table S4: Antibacterial activity of five different cultivars of date pit extracts.
