# Supplementary material for: Antibacterial and Immunostimulatory Effects of Raziz Date Palm Pits in Streptococcus agalactiae-Infected Red Hybrid Tilapia
Source: Biology (Basel). 2025 Oct 3;14(10):1356. doi: 10.3390/biology14101356 (PMC12561124; doi:10.3390/biology14101356)
Supplement: Supplementary file 1 [file biology-14-01356-s001.zip › biology-3839963-supplementary.pdf]

Supplementary data

Table S1. Primer sequences and conditions of PCR cycle

No.	Primer sequence (5′ – 3′)	Target gene	Amplicon size	Accession number
1.	Fw: TGAGTGCAGAAGGGGAGAGT	16S r-RNA	892 bp	AE009948.1
	Rv: GAAAGGAGGTGATCCAGCCG			
2.	Fw: GTCAAAGGGACTAGCACC ACA	Ia *cps*	211 bp	CP000114.1
	Rv: TGGCTCCGTAAAGATTTGTCCA			
3.	Fw: AAGGACACAACCCCTACTGC	Ib *cpsG*	249 bp	FO393392.1
	Rv: AGGAGGAATTTTCGCTTTAACCC			
4.	Fw: TATTGGCCTTCAGACGAGCG	II *cpsH*	208 bp	LT671985.1
	Rv: AGCGTAACTTTGACACACCA			
	Fw: CAGGTGTTTACAGGGACGGA	II *cpsH*	962 bp	LT671985.1
	Rv: TGGCTCGCGTATATAATCTTCGG			
	Fw: CACGTTGATGCCAAAAGTGAT	II *cpsP*	422 bp	LT671985.1
	Rv: CCCTTACGAAGAGGAGGTAGG			

	Fw: TGAAGAGTTCCGAAATGAAGTCC	II *cpsQ*	309 bp	LT671985.1
	Rv: GCTTTTTGTCCTATTTTCACCCC			
5.	Fw: GATCTATTTCCTCAAAGAGCGTG	III *cpsH*	213 bp	AL766849_1
	Rv: GACAGGGTATAAGGATTCCATCAAG			

PCR amplification conditions

No.	Step	Temperature (°C)	Time (minutes)
1.	Initial denaturation	95	10
2.	Denaturation	95	1
3.	Annealing	53	0.5
4.	Elongation	72	1 min/kb
5.	Final extension	72	5

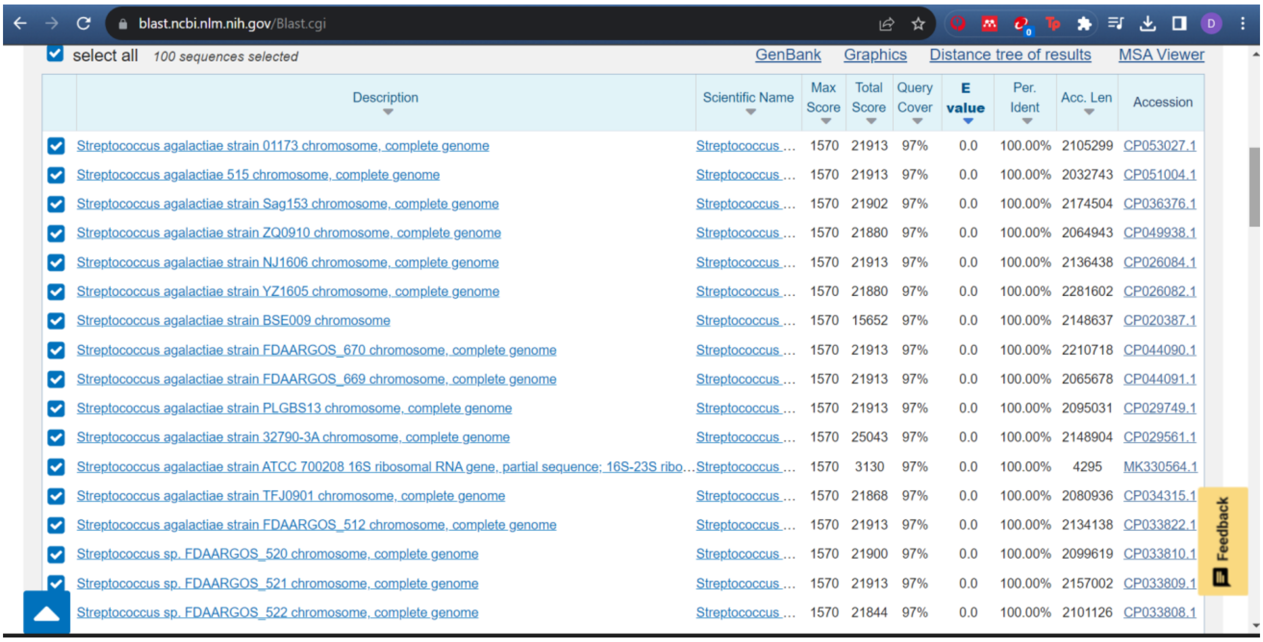

Description	Scientific Name	Max Score	Total Score	Query Cover	E value	Per. Ident	Acc. Len	Accession
Streptococcus agalactiae strain 01173 chromosome, complete genome	Streptococcus ...	1570	21913	97%	0.0	100.00%	2105299	CP053027.1
Streptococcus agalactiae 515 chromosome, complete genome	Streptococcus ...	1570	21913	97%	0.0	100.00%	2032743	CP051004.1
Streptococcus agalactiae strain Sag153 chromosome, complete genome	Streptococcus ...	1570	21902	97%	0.0	100.00%	2174504	CP036376.1
Streptococcus agalactiae strain ZQ0910 chromosome, complete genome	Streptococcus ...	1570	21880	97%	0.0	100.00%	2064943	CP049938.1
Streptococcus agalactiae strain NJ1606 chromosome, complete genome	Streptococcus ...	1570	21913	97%	0.0	100.00%	2136438	CP026084.1
Streptococcus agalactiae strain YZ1605 chromosome, complete genome	Streptococcus ...	1570	21880	97%	0.0	100.00%	2281602	CP026082.1
Streptococcus agalactiae strain BSE009 chromosome	Streptococcus ...	1570	15652	97%	0.0	100.00%	2148637	CP020387.1
Streptococcus agalactiae strain FDAARGOS_670 chromosome, complete genome	Streptococcus ...	1570	21913	97%	0.0	100.00%	2210718	CP044090.1
Streptococcus agalactiae strain FDAARGOS_669 chromosome, complete genome	Streptococcus ...	1570	21913	97%	0.0	100.00%	2065678	CP044091.1
Streptococcus agalactiae strain PLGBS13 chromosome, complete genome	Streptococcus ...	1570	21913	97%	0.0	100.00%	2095031	CP029749.1
Streptococcus agalactiae strain 32790-3A chromosome, complete genome	Streptococcus ...	1570	25043	97%	0.0	100.00%	2148904	CP029561.1
Streptococcus agalactiae strain ATCC 700208 16S ribosomal RNA gene, partial sequence; 16S-23S ribo...	Streptococcus ...	1570	3130	97%	0.0	100.00%	4295	MK330564.1
Streptococcus agalactiae strain TFJ0901 chromosome, complete genome	Streptococcus ...	1570	21868	97%	0.0	100.00%	2080936	CP034315.1
Streptococcus agalactiae strain FDAARGOS_512 chromosome, complete genome	Streptococcus ...	1570	21913	97%	0.0	100.00%	2134138	CP033822.1
Streptococcus sp. FDAARGOS_520 chromosome, complete genome	Streptococcus ...	1570	21900	97%	0.0	100.00%	2099619	CP033810.1
Streptococcus sp. FDAARGOS_521 chromosome, complete genome	Streptococcus ...	1570	21913	97%	0.0	100.00%	2157002	CP033809.1
Streptococcus sp. FDAARGOS_522 chromosome, complete genome	Streptococcus ...	1570	21844	97%	0.0	100.00%	2101126	CP033808.1

Figure S1. BLAST result indicating the isolate is *Streptococcus agalactiae*.

Table S2. Concentration of commercial antibiotics tested and expected Zones of Inhibition

Antibiotics	Concentration (μg/mL)			Zone of inhibition (mm)		
	S	I	R	S	I	R
**Ampicillin**	≤ 0.25	-	-	≥ 24	-	-
**Chloramphenicol**	≤ 4	8	≥ 16	≥ 21	18-20	≤17
**Tetracycline**	≤ 2	4	≥ 8	≥ 23	19-22	≤ 18

Table S3. Zones of Inhibition for commercial antibiotics tested

Antibiotics	Concentration tested (μg/mL)			Zone of inhibition[a] (mm)		
	S	I	R	S	I	R
**Ampicillin**	0.25	-	-	0	-	-
**Chloramphenicol**	4	8	16	0	0	4 ± 2
**Tetracycline**	2	4	8	0	2 ± 2	2.333 ± 2.333

.

Table S4. Antibacterial activity of five different cultivars of date pit extracts

	Extract tested		
**Date pits**	Water extract	Methanol extract	Chloroform extract
**Bouman**	Presence of zone of inhibition at concentration of 33.3%.	Presence of zone of inhibition at a concentration of 2 g/mL.	No activity for all 3 tested concentrations.
**Fardh**	Presence of zone of inhibition at a concentration of 33.3%.	No activity for all 3 tested concentrations.	Presence of zone of inhibition at a minimum concentration of 1 g/mL.

**Khallas**	Presence of zone of inhibition at a minimum concentration of 16.5%.	No activity for all 3 tested concentrations.	Presence of zone of inhibition at a concentration of 2 g/mL.
**Lulu**	Presence of zone of inhibition at a concentration of 33.3%.	No activity for all 3 tested concentrations.	No activity for all 3 tested concentrations.
**Raziz**	Presence of zone of inhibition at a minimum concentration of 16.5%.	Presence of zone of inhibition at a minimum concentration of 1 g/mL.	Presence of zone of inhibition at a concentration of 2 g/mL.
